# HIV-1 subtype distribution in the Gambia and the significant presence of CRF49_cpx, a novel circulating recombinant form

**DOI:** 10.1186/1742-4690-7-82

**Published:** 2010-10-09

**Authors:** Thushan I de Silva, Roxanne Turner, Stéphane Hué, Roochi Trikha, Carla van Tienen, Clayton Onyango, Assan Jaye, Brian Foley, Hilton Whittle, Sarah L Rowland-Jones, Matthew Cotten

**Affiliations:** 1 Medical Research Council (UK) Laboratories, Atlantic Road, PO Box 273, Fajara, The Gambia; 2 MRC/UCL Centre for Medical Molecular Virology, Division of Infection and Immunity, University College London, UK; 3 Weatherall Institute of Molecular Medicine, Medical Research Council Human Immunology Unit, John Radcliffe Hospital, Oxford, UK; 4 Theoretical Biology & Biophysics, Los Alamos National Laboratory, Los Alamos, NM 87545, USA

## Abstract

**Background:**

Detailed local HIV-1 sequence data are essential for monitoring the HIV epidemic, for maintaining sensitive sequence-based diagnostics, and to aid in designing vaccines.

**Results:**

Reported here are full envelope sequences derived from 38 randomly selected HIV-1 infections identified at a Gambian clinic between 1991 and 2009. Special care was taken to generate sequences from circulating viral RNA as uncloned products, either by limiting dilution or single genome amplification polymerase chain reaction (PCR). Within these 38 isolates, eight were subtyped as A and 18 as CRF02_AG. A small number of subtype B, C, D viruses were identified. Surprising, however, was the identification of six isolates with subtype J-like envelopes, a subtype found normally in Central Africa and the Democratic Republic of the Congo (DRC), with *gag *p24 regions that clustered with subtype A sequences. Near full-length sequence from three of these isolates confirmed that these represent a novel circulating recombinant form of HIV-1, now named CRF49_cpx.

**Conclusions:**

This study expands the HIV-1 sequence database from the Gambia and will provide important data for HIV diagnostics, patient care, and vaccine development.

## Background

Current data on the HIV epidemic in the Gambia are lacking. The most recent published data on HIV prevalence in the general population are from a nationwide perinatal clinic survey in 2000-2001 and indicate a low, but possibly increasing prevalence of HIV-1 infection in the country [1]. More recent data from the Medical Research Council Laboratories Genitourinary medicine (GUM) clinic indicate that although HIV-2 infection frequency is declining in patients attending the clinic, the HIV-1 prevalence rose from 4.2% in 1988 to 17.5% in 2003 [2]. Information on the genetic diversity of the local HIV-1 subtypes and genetic variety is also not abundant. The Los Alamos HIV Database (LAHDB) [3] currently lists only 31 sequence entries reporting subtype information from the Gambia, while the surrounding country Senegal has 840 reports, neighboring Mali has 392, and Guinea Bissau has 290. Detailed sequence data are required to correctly document the AIDS epidemic, to trace the infection history, monitor changes in infection patterns and to maintain sensitive and accurate viral diagnostics. Furthermore, whether future HIV-1 vaccine strategy is based on immunogens optimized for local strains, or recently described 'global' mosaic vaccines that maximize coverage across HIV-1 strains worldwide [4,5], ongoing documentation of HIV-1 sequence diversity is crucial. The current study was an attempt to improve the local HIV-1 sequence database.

Reported here are the full envelope gene (*env*) sequences derived from 38 HIV-1 infections identified at a Gambian clinic between 1991 and 2009, as well as three near full-genome sequences from a novel complex circulating recombinant form (CRF) identified in the study. The length of *env *sequence derived from each patient (approximately 2500 bp) allowed a robust determination of HIV-1 subtype.

## Methods

### Patient selection

The viral sequences were obtained from patients attending the Genito-Urinary Medicine (GUM) clinic in Fajara, the Gambia, who had archived plasma samples available. Patient selection was based on two criteria (see below) and PCR was attempted on a total of 53 patient samples: the first group of 33 patients were selected at random from all those enrolled in the cohort with a CD4 count of ≥ 28% at diagnosis (these criteria were applied in order to use the amplified products for a concurrent study). The second group of five patients were selected at random from individuals who had recently been diagnosed with advanced HIV infection and started on antiretroviral therapy (ART); these patients therefore had lower CD4 counts (median CD4% of 13 for the ART group, 35 for the non-ART group). Additional patient details are given in Table 1. For this second group of patients, the last blood sample before initiating ART was used as the source of virus.

**Table 1:** Cohort Summary

ID	Sex	Age at diagnosis	Ethnicity	ART	Year of diagnosis	Subtype
N006909	F	27	Wolof	no	1997	A

N009845	F	42	Jola	no	2000	A

N040736	F	29	Mandinka	no	2005	A

N057856	F	25	Mandinka	no	2009	A

N058579	M	49	Mandinka	yes	2009	A

N059096	F	35	Jola	yes	2009	A

N33456	M	52	Fula	no	2005	A

N75698	F	50	Manjago	no	1994	A

N004445	M	37	Jola	no	1999	CRF02_AG

N010897	M	25	Mandika	no	1999	CRF02_AG

N011064	F	34	Mandinka	no	2000	CRF02_AG

N016805	F	35	Jola	no	2002	CRF02_AG

N017561	F	32	Mandinka	no	2002	CRF02_AG

N018622	M	18	Mandinka	no	2000	CRF02_AG

N022314	F	32	Mandinka	no	2003	CRF02_AG

N041366	M	40	Mandinka	no	2006	CRF02_AG

N047046	F	60	Mandinka	no	2006	CRF02_AG

N056537	F	24	Mandinka	no	2008	CRF02_AG

N058521	M	64	Wolof	no	2008	CRF02_AG

N058628	F	26	Wolof	yes	2009	CRF02_AG

N059677	F	30	Other	yes	2009	CRF02_AG

N180032	F	30	Fula	no	1995	CRF02_AG

N32458	F	24	Mandinka	no	2003	CRF02_AG

N32468	F	26	Wolof	no	2004	CRF02_AG

N36165	F	25	Jola	no	2005	CRF02_AG

N73487	F	25	Fula	no	1993	CRF02_AG

N059733	M	39	Wolof	yes	2009	B

N005312	F	22	Mandinka	no	1991	C

N025015	F	30	Mandinka	no	2003	C

N025567	M	34	Fula	no	1996	C

N001823	F	20	Jola	no	1998	D

N73603	F	23	Serahuli	no	1993	D

N001605	F	22	Jola	no	1998	CRF49_cpx

N005284	F	20	Mandinka	no	1999	CRF49_cpx

N018380	M	29	Manjago	no	2002	CRF49_cpx

N024017	F	29	Mandinka	no	1998	CRF49_cpx

N026677	F	37	Manjago	no	2002	CRF49_cpx

N28353	F	29	Serahuli	no	1996	CRF49_cpx

### Viral RNA Extraction

Viral RNA was extracted from 200 μl of plasma diluted in 800 μl of RNase free water using the QIAamp Ultrasens Viral RNA Extraction Kit (QIAGEN) with final elution into 60 μl. Each sample was loaded on a single column and washed according to the manufacturer's protocol.

### Amplification of full-length HIV-1 *env*

Reverse transcription and the first round of a nested PCR reaction were performed in single reaction. Each 25 μl RT-PCR reaction contained the following mix: 1 × PCR buffer Titan One Tube System (Roche Applied Science), 2.5 mM MgCl2, 400 nM dNTP mix, 0.1 μM of primers O_*envf *and O_*envr*, 0.208 U/μl RNase inhibitor, 1 μl of the Titan One Tube enzyme mix and 5 μl of extracted RNA. Reverse transcription proceed at 45°C for 45 min. followed by 95°C for 3 min, 10 cycles of 94°C (30 sec), 56°C (30 sec), 68°C (3 min), followed by 30 cycles of 94°C (30 sec), 56°C 30 sec), 68°C (3 min) plus 5 sec time extension at 68°C after each round and a final extension of 7 min at 68°C. The inner (nested) PCR reactions used 1 μl of the first-round RT-PCR product in 50 μl containing: 1 × Buffer (with 1.5 mM MgCl_2 _final concentration), 0.05U/μl Expand HiFi Plus polymerase (Roche Applied Science), 400 nM dNTP mix, 0.25 μM of primers MO130 and MO147. Amplification was conducted at 95°C for 3 min followed by 40 cycles of 94°C (15 sec), 56°C (30 sec), 72°C (3 min), and a final extension of 7 min at 72°C. The PCR products were resolved on a 1% agarose (Tris-Borate EDTA, TBE) gel, DNA was visualized by ethidium bromide staining and the 2.5 kb product purified using the MinElute Gel Extraction Kit (QIAGEN).

### Amplification of HIV-1 p24

Reverse transcription and the first round of a nested PCR reaction were performed in single reaction. Each 50 μl RT-PCR reaction contained the following mix: 1 × PCR buffer Titan One Tube System (Roche Applied Science), 2.5 mM MgCl_2_, 200 nM dNTP mix, 0.5 μM of primers MO042 or MO024 (alternate outer forward) and MO044, 0.208 U/μl RNase inhibitor, 1 μl of the Titan One Tube enzyme mix and 10 μl of extracted RNA. Reverse transcription proceed at 50°C for 30 min, followed by 95°C for 3 min, 40 cycles of 94°C (30 sec), 54°C (30 sec), 72°C (1 min) and a final extension of 7 min at 72°C. The inner (nested) PCR reactions used 1 μl of the first-round RT-PCR product in 50 μl containing: 1 × Buffer (with 1.5 mM MgCl2 final concentration), 0.05U/μl Expand HiFi Plus polymerase (Roche Applied Science), 400 nM dNTP mix, 0.5 μM of primers MO043 and MO045. Amplification was conducted at 95°C for 3 min followed by 40 cycles of 94°C (30 sec), 56°C (30 sec), 72°C (1 min), and a final extension of 7 min at 72°C. The PCR products were resolved on a 1% agarose (Tris-Borate EDTA, TBE) gel, DNA was visualized by ethidium bromide staining and the product was purified using the MinElute Gel Extraction Kit (QIAGEN).

### Amplfication of near full-length HIV-1 genomes

In addition to *env *and p24 fragments, near full-length genome sequence was obtained by amplifying three further fragments: (A) 5' LTR to *gag *p24, (B) *gag *p24 to *env  *and (C) *env *to 3' LTR. For fragment (A), reverse transcription and the first round of a nested PCR reaction were performed in single reaction. Each 25 μl RT-PCR reaction contained the following mix: 1 × PCR buffer Titan One Tube System (Roche Applied Science), 2.5 mM MgCl2, 400 nM dNTP mix, 0.5 μM of primers MO034 and MO191, 0.208 U/μl RNase inhibitor, 1 μl of the Titan One Tube enzyme mix and 5 μl of extracted RNA. Reverse transcription proceed at 50°C for 30 min, followed by 95°C for 3 min, 40 cycles of 94°C (30 sec), 54°C (30 sec), 72°C (1 min) and a final extension of 7 min at 72°C. The inner (nested) PCR reactions used 1 μl of the first-round RT-PCR product in 50 μl containing: 1 × Buffer (with 1.5 mM MgCl2 final concentration), 0.05U/μl Expand HiFi Plus polymerase (Roche Applied Science), 400 nM dNTP mix, 0.5 μM of primers MO024 and MO192. Amplification was conducted at 95°C for 3 min followed by 40 cycles of 94°C (30 sec), 56°C (30 sec), 72°C (1 min), and a final extension of 7 min at 72°C. Fragment (C) was amplfied with a nested PCR on products obtained with primers O_*envf *and O_*envr *as described above. The inner (nested) PCR reactions and conditions were identical to those used above for fragment (A), but using primers MO193 and MO194. For fragment (B), reverse transcription was performed in a 20 μl reaction containing 1× Qiagen LongRange RT buffer, 1 mM mix of each dNTP, 1 μM of primer MO187, 0.04 U/μl RNase inhibitor, 1 μl LongRange Reverse Transcriptase (Qiagen) and 10 μl of extracted RNA. Reactions were incubated at 42°C for 90 minutes followed by 85°C for 5 minutes. Each 50 μl first round PCR contained the following: 1× Expand Long Template (Roche Applied Science) buffer 1 (with 1.75 mM MgCl2 final concentration), 400 nM dNTP mix, 0.3 μM of primers MO186 and MO187, 0.75 μl of the Expand Long Template enzyme mix and 5 μl of cDNA template. PCR conditions were as follows: 94°C for 2 min, 10 cycles of 94°C (10 sec), 56°C (30 sec), 68°C (4 min), followed by 30 cycles of 94°C (10 sec), 56°C (30 sec), 68°C (4 min) plus 20 sec time extension at 68°C after each round and a final extension of 7 min at 68°C. The inner (nested) PCR used 1 μl of the first round PCR product in 50 μl containing 1 × Expand Long Template (Roche Applied Science) buffer 1 (with 1.75 mM MgCl2 final concentration), 400 nM dNTP mix, 0.5 μM of primers MO188 and MO189 and 0.75 μl of the Expand Long Template enzyme mix. Amplification was conducted using the same conditions as described above for the first round PCR.

### Limiting dilution PCR and Single Genome Amplification

All *env *fragments were initially amplified using bulk PCR conditions on undiluted template and sequencing was carried out as described below for the highly variable V1/V2 region, followed by the entire *env *fragment if no double peaks were observed. In those samples showing multiple peaks in the V1/V2 region, the cDNA was then amplified using two different dilution methods in order to obtain amplification from single genomes. Both methods involved diluting the cDNA and running a standard PCR. First, three-fold limiting dilution of a single cDNA sample (reverse transcribed using the Titan One Tube RT-PCR reaction mix, for 45 min at 45°C) was carried out (from 1:3 to 1:243), followed by the standard first round and nest PCR conditions as described above. The highest dilution at which the *env *fragment amplification was successful was chosen for sequencing. If the V1/V2 region still contained multiple sequences, single genome amplification was carried out with a modified protocol to that described in the literature [6]. Briefly, three-fold dilution of cDNA was carried out with nine replicates per dilution (starting at the highest dilution at which the single sample limiting dilution PCR was successful), followed by the standard first round and nest PCR conditions as described above. An amplified *env *from the dilution where only one or two replicates yielded a positive PCR reaction (i.e. <30% of replicates positive [6]) was selected for sequencing and purified using the MinElute Gel Extraction Kit (QIAGEN).

### Sequencing strategy

The full-length *env *products were sequenced using a set of overlapping reactions. The internal nested primers, MO130 and MO147, were used as the 5' most and 3' most primers for sequencing. An additional six primers were designed to generate eight contigs covering the full *env *sequence (see Table 2 for details). Sequencing primers were designed to hybridize to conserved regions ca. 600-800 bp apart using a collection of 30 West African sequences from the LAHDB plus the reference HIV-1 HXB2. The p24 PCR products were sequenced using internal nested primers MO043 and MO045. Additional fragments required to assemble near full-genome sequence were sequenced as follows: fragments (A) and (C) were sequenced with internal nested primers MO024/MO192 and MO193/MO194 respectively. For fragment (B), internal nested primers, MO188 and MO189 were used as the 5' most and 3' most primers, along with 13 additional primers designed as described above to span the entire region from *gag *to *env *(see Table 2 for details). All primers for PCR and sequencing were synthesized by Metabion (Metabion International AG, Lena-Christ-Str. 44/I, 82152 Martinsried, Germany, [7]). Sequencing reactions were carried out by Macrogen [8].

**Table 2:** Primers used in this work

Name	Function	**Position in HXB2**^**2**^	Sequence (5' to 3')
O_*envf*	*env *PCR OF^1^	5964-5984	TYTCCTATGGCAGGAAGAAGC

O_*envr*	*env *PCR OR	9096-9074	TAACCCWTCCAGTCCCCCCTTTT

MO130	*env *PCR IF	6207-6228	GAGCAGAAGACAGTGGCAATGA

MO147	*env *PCR IR	8834-8810	CATCCMACTATRCTRCTTTTTGACC

MO150	*env *sequencing	6976-6955	ATTCCATGTGTACYTTGTACTG

MO151	*env *sequencing	6859-6880	CAATTCCCATACATTATTGTGC

MO152	*env *sequencing	7668-7647	CACTTCTCCAATTGTCCRTCAT

MO153	*env *sequencing	7516-7537	GACAAGCAATGTATGCCCCTCC

MO154	*env *sequencing	8241-8220	ACCAATTCCACAYACTTGCCCA

MO155	*env *sequencing	8050-8072	CTGGAACKCTAGTTGGAGTAAT

MO042	*gag *p24 OF-1	890-909	TAGTATGGGCAAGCAGGGAG

MO024	*gag *p24 OF-2	508 - 527	AACCCACTGCTTAAGCCTCA

MO044	gag p24 OR	2272-2252	TGCCAAAGAGTGATTTGAGGG

MO043	*gag *p24 IF	1048-1067	TGYGTRCATCAAARGATAGA

MO045	*gag *p24 IR	2118-2101	CCCCTTGYTGGAAGGCCA

MO034	5' LTR to *gag *p24 OF	478 - 479	TGAGCCTGGGAGCTCTCTG

MO186	p24 to *env *OF	1958 - 1985	TTAARTGTTTCAACTGTGGCAAAGAAGA

MO187	p24 to *env *OR	6420 - 6445	CAAGCATGKGTAGCCCAGAYATTATG

MO188	p24 to *env *IF	2034 - 2060	ATGTGGGAARGARGGACACCAAATGAA

MO189	p24 to *env *IR	6335 - 6360	TCCACACAGGTACCCCATAATAGACT

MO191	5' LTR to *gag *p24 OR	832 - 859	AATGCTGWRAACATGGGTATTACTTCTG

MO192	5' LTR to *gag *p24 IR	786 - 814	TCTATTACTTTYACCCATGCATTTAAAGT

MO193	*env *to 3' LTR IF	7922 - 7944	CAGACCCTTATCCCAAACCCAAC

MO194	*env *to 3'LTR IR	8606 - 8629	CCCCCCTTTTCTTTTAAAAAGWRGC

AJB-1R	p24 to *env *sequencing	2239 - 2262	TATGGATTTTCAGGYCCAATTYTTG

AJB-2F	p24 to *env *sequencing	2036 - 2058	GCCCAAARGTTAAACAATGGCCA

AJB-3R	p24 to *env *sequencing	2846 - 2871	TTCTGTATRTCATTGACAGTCCAGCT

AJB-4F	p24 to *env *sequencing	2741 - 2765	ACACCAGAYAARAARCATCAGAAAG

AJB-5R	p24 to *env *sequencing	3585 - 3610	GATTCCTAATGCATACTGTGAGTCTG

AJB-6F	p24 to *env *sequencing	3585 - 3610	CAGACTCACAGTATGCATTAGGAATC

AJB-7R	p24 to *env *sequencing	3722 - 3750	ACTAATTTATCTACTTGTTCATTTCCGCC

AJB-8R	p24 to *env *sequencing	4357 - 4383	ATGTCTAYTATTCTTTCCCCTGCACTG

AJB-9F	p24 to *env *sequencing	4196 - 4219	ATTCCCTACAATCCCCAAAGMCARG

AJB-10F	p24 to *env *sequencing	4609 - 4633	TGATTGTGTGGCARGTAGACAGGAT

AJB-11R	p24 to *env *sequencing	4830 - 4854	TCCATTCTATGGAGACYCCMTGACC

AJB-12R	p24 to *env *sequencing	5498 - 5521	TGCCATAGGARATGCCTAAGCCYTT

AJB-13F	p24 to *env *sequencing	5498 - 5521	AARGGCTTAGGCATYTCCTATGGCA

^1^Abbreviations:OF, outer forward;, OR, outer reverse; IF, inner forward, IR, inner reverse.

^2 ^HXB2 numbering is based on sequence with accession number K03455

### Assembling full-length *env*, p24 and near full-genome sequences

For all samples, the sequencing chromatograms were carefully inspected for sites of ambiguous sequence. All reliable sequence data were assembled using the BioEdit Sequence Alignment Editor [9,10] and aligned using the Cap Contig Assembly program. For each assembled sequence, the open reading frame (ORF) was established using alignments with HXB2 *env *and the ORF finder in the Sequence Manipulation Suite [11,12]. In areas where premature stop codons appeared, the sequence chromatograms were re-examined to determine if miscalled nucleotides in the region could account for the loss of the open reading frame. Such errors were manually corrected to give full reads of the respective sequence.

All sequences described in this manuscript have been deposited in GenBank with the following accession numbers: Envelopes (n = 35): HQ385442 - HQ385476; CRF49 genomes (n = 3): HQ385477 - HQ385479; 3 extra p24 sequences from presumed CRF49 isolates (n = 3): HQ385480 - HQ385482.

### HIV-1 subtyping and phylogenetic analyses

HIV-1 subtype was assigned to each completed sequence in the following manner. *Env *DNA sequences from each subject, along with the HIV-1 subtype reference set (2005) obtained from the LAHDB, additional CRF02_AG sequences DJ263 (Djibouti), MP1211 (Senegal), MP1213 (Senegal) (accession numbers AB485634, AJ251056 and AJ251057 respectively) and additional A3 *env *sequences from Senegal (DD1579, DDJ360, DDJ362 and DDJ364; accession numbers AY521629, AY521630, AY521632 and AY521633 respectively) [13,14] were aligned using CLUSTALW2 [15,16]. All alignments were inspected and edited manually using Se-Al (Sequence Alignment editor, v2.0a11, Rambaut, A. Department of Zoology, University of Oxford, UK), and ambiguous regions with multiple indels were deleted. Phylogenetic trees were constructed with the program PAUP* version 4.0b10 [17] using a maximum likelihood (ML) approach [18]. The trees were reconstructed under the General Time Reversible model of nucleotide substitution [19], with proportion of invariable sites and substitution rate heterogeneity. The statistical robustness of the ML topologies was assessed by bootstrapping with 1000 replicates using the neighbour-joining method. The software Inkscape [20] was used to color code and label the trees.

### Phylogenies of *env*, p24 and near full-length sequences from CRF49_cpx isolates

*Env *fragments from six individuals designated as subtype J-like using the phylogenetic analyses described above were further aligned with all available subtype J *env  *sequence of approximately 1200 bp or above in length in the LAHDB: SE92809 (AF082394), SE9173 (AF082395), MBTB4 (AJ401046), KTB147 (AJ401041), MBS41 (AJ4010145), VLGCJ1 (AY669766), VLGCJ2 (AY669767), 98BW21.17 (AF192135), GM4 (U33099), GMB22 (AJ276694) and GMB24 (AJ276695). All sequences were trimmed to the length of the shortest sequence, thus an alignment containing 1125 bp fragments were used to build a subtype J *env *phylogenetic tree using the methodology described above.

The p24 sequence from these six individuals were also aligned with HIV-1 subtype A and CRF02_AG reference isolates from the LAHDB (2005) subtype reference set [3], additional *gag *sequence from three CRF02_AG isolates SE7812 (AF107770), MP1211 (AJ251056), MP1213 (AJ251057), three A3 Senegalese isolates DDJ360 (AY521630), DDI579 (AY521629), DDJ369 (AY521631) [13,14], additional subtype A1 isolates SE7535 (AF069671), SE8891 (AF069673), SE8131 (AF107771), SE8538 (AF069669). and the DRC isolates MBTB4 (AJ404293), KCC2 (AM000053), KTB13 (AM000054) and KTB035 (AM000055). A phylogenetic tree was reconstructed with the methodology described above.

Near full-genome sequences obtained from three of these isolates were aligned with the 2008 LAHDB subtype reference set and isolates 98 BW21.17 (AF192135), DDJ360 (AY521630), DDI579 (AY521629) and DDJ369 (AY521631). Bayesian Markov chain Monte Carlo (MCMC) phylogenies were estimated under the General Time Reversible model of nucleotide substitution with gamma-distributed rate heterogeneity, using the program MRBAYES version 3.1.2. [21]. The Bayesian MCMC search was set to 1,500,000 iterations with trees sampled every 100 th generations. A maximum clade credibility tree (MCCT) was selected from the sampled posterior distribution with the programTreeAnnotator version 1.5.2 http://beast.bio.ed.ac.uk/, after discarding trees corresponding to a 10% burnin. The MCCT Tree was edited with the program FigTree version 1.1.2.

### Characterization of subtype recombination in CRF49_cpx

Simplot and bootscan analyses of near full-genome isolates N18380_GM, N26677_GM and N28353_GM were performed using Simplot [22]. Pure subtypes A through K were included (and in a second analysis, isolate 98BW21.17 added) and the alignment was globally gap stripped. Sliding window was set to 400 bp and increments set to 50 bp. Bootscanning was performed using the neighbour-joining method, using the Kimura (two-parameter) distance model and 100 bootstrap replicates for each sliding window. The transition/traversion ratio was set to 2.0. For each CRF49_cpx sequence, markers were placed at breakpoints between subtypes and an alignment of each fragment used to construct phylogenetic trees using the maximum likelihood methodology (and bootstrapping with 1000 replicates using the neighjour-joining method) described above. The HIV Sequence Locator tool at the LAHDB was used to assign HXB2 numbering to each fragment and the Recombinant HIV-1 Drawing Tool (also at the LAHDB) utilised to construct a recombinant map of CRF49_cpx representing a consensus of breakpoints across the three full genomes.

## Results and Discussion

### Description of the Cohort

The majority of the subjects was female (n = 28, 74%); a higher percentage of women attending the GUM clinic in Gambia has been reported and may be due to changes in referral policies and sex-specific differences in health-care seeking behaviour [2]. The median age at diagnosis was 29.5 years. The ethnic composition of the cohort was largely similar to the Gambian general population with Mandinka 42% (42% in general population), Fula 11 (18), Wolof 13 (16), Jola 18 (10), Serahuli 5 (9), Manjago 8 (not listed) and other groups 3 (4). The numbers in parentheses are from the 2003 census data [23]. The number of Jola subjects (18.4%) was noticeably higher than the general population (10%).

### Virus subtyping

**Figure 1:**
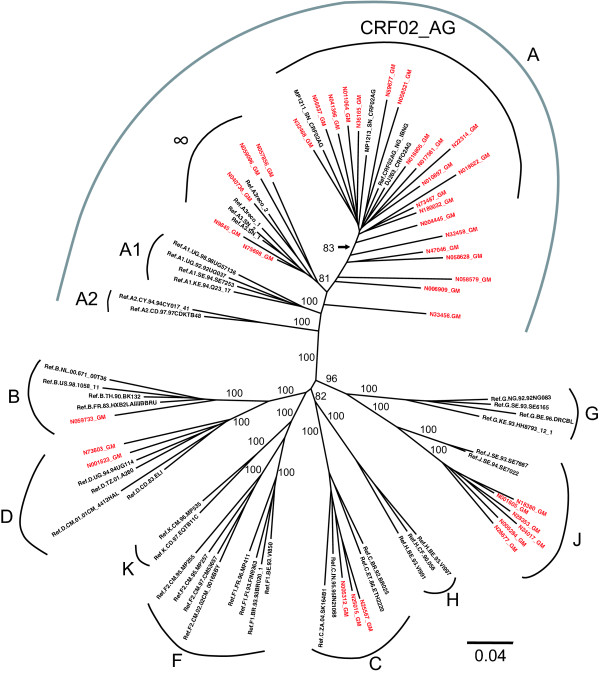
**Phylogenetic classification of 38 new Gambian HIV-1 full-length env sequences (highlighted in red), along with reference subtypes and additional subtype A sequences (CRF02_AG and Senegalese A3 variants)**. The full Los Alamos HIV Database (2005) subtype reference set was initially used to construct the tree, but all CRFs other than CRF02_AG have been omitted here for clarity. The phylogenetic tree was constructed using a maximum likelihood method [18], under the General Time Reversible model of nucleotide substitution [19], with proportion of invariable sites and substitution rate heterogeneity. Bootstrap percentiles above 70% from 1000 replications (using the neighbor-joining method) are shown at the corresponding branches defining major grouping of sequences. Five of the new Gambian sequences cluster with the Senegalese A3 variant sequences with a bootstrap support of 81 (∞). Branch lengths represent the number of substitutions per nucleotide sites.

The subtype assignment of the 38 env sequences was obtained by aligning the sequences with LAHDB HIV-1 (2005) subtype reference sequences (which includes approximately four reference sequences from each relevant subtype), along with an additional three CRF02_AG and four A3 sequences (two from A3/CRF02_AG recombinants) as described above and constructing a maximum likelihood tree. As none of the new Gambian *env *sequences clustered with currently known recombinant forms other than CRF02_AG, for clarity Fig. 1 displays reference isolates from pure subtypes and CRF02_AG only.

Five of the new Gambian sequences (N057856_GM, N059096_GM, N9845_GM, N75698_GM and N040736_GM) clustered with the Senegalese A3 (DDJ360, DD1579) and A3/CRF02_AG recombinant (DDJ364, DDJ362) sequences [13,14] with a bootstrap support of 81% (see Fig. 1 cluster denoted by ∞). Given the regional frequency of A3-like viruses, their occurrence in Gambia is not unexpected. Four isolates (N59677_GM, N058521_GM, N22314_GM and N018622_GM) clustered with reference and Gambian CRF02_AG sequences (bootstrap support 83%), although it can be difficult to distinguish subtype A (A1, A2, A3) from CRF02_AG isolates based in *env *alone as this region is largely subtype A derived in CRF02_AG [24]. An additional four isolates did not form significant clusters (N32458_GM, N47046_GM, N058628_GM and N006909_GM). Thus these data do not support the existence of a Gambian-specific AG sub-subtype. From this analysis, it appears that the heterogeneity within the global CRF02_AG subgroup is equally reflected within the Gambian AG viruses. It is clear that the subtype A *env *sequences from circulating Gambian strains are distinct from both A1 and A2 reference isolates in the LAHDB, and more closely related to Senegalese A3 or CRF02_AG isolates.

In addition to the A and AG like isolates, the novel viruses include a single subtype B (N059733_GM), three subtype C isolates (N005312_GM, N25667_GM, N025015_GM) and two subtype D isolates (N73603_GM, N001823_GM) clustering with high bootstrap values within the reference isolate clusters for these subtypes (Fig. 1). Of special interest were six isolates (N18380_GM, N001605_GM, N24017_GM, N28353_GM, N005284_GM and N26677_GM) forming a monophyletic cluster within the subtype J branch (bootstrap value of 100%, see Fig. 1 and below).

An additional consideration was raised by the recent analysis concluding that CRF02_AG is more likely to be a pure subtype and the precursor to subtype G, which may in turn be a recombinant derived from subtypes CRF02_AG and J [25]. This history could account for the high prevalence of CRF02_AG in West Africa and may account for local differences (for example between Senegal and Gambia) in the prevalence of subtype G and J viruses. A more recent analysis has however questioned these claims and suggested that CRF02_AG did indeed arise as a result of recombination events that occurred early in the divergence between subtype A and G [26].

### Isolates with subtype J-like *env *have subtype A *gag *regions

**Figure 2:**
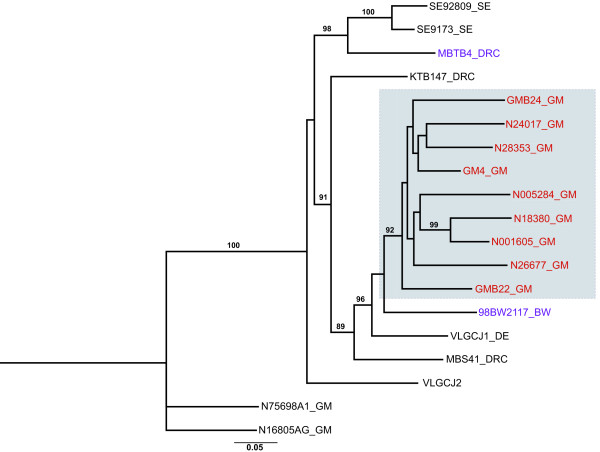
**Phylogenetic tree with all available subtype J-like *env *Gambian isolates (red), including the three older isolates GM4, GM22 and GM24, and other subtype J *env *sequences from the Los Alamos HIV Database**. MBTB4 and 98BW21.17 (in purple) are subtype A *gag */J *env *recombinants described from outside the Gambia (DRC and Botswana respectively). The Gambian subtype J-like *env *monophyletic cluster is boxed. SE92809 and SE9173 are the two subtype J reference strains (From DRC, isolated in Sweden). The phylogenetic tree was reconstructed as in Fig. 1 and bootstrap percentiles above 70% from 1000 replications (using the neighbour-joining method) are shown. The tree is rooted by outgroups formed by subtype A1 and CRF02_AG *env *fragments from the Gambia (N75698A1_GM and N16805_GM). Branch lengths are expressed as the number of substitutions per nucleotide sites.

Three previous Gambian HIV-1 samples, GM4 (U33099), GM5 and GM7, were reported to be distinct from the pure HIV-1 subtypes A to G known at the time [27] when the J subtype had not yet been defined. GM4 is described in the LAHDB as a subtype CGJ mosaic, although phylogenetic analyses suggest that it is subtype J-like in *env *[28]. Since that time, two additional Gambian J-like *env *sequences were reported (GMB22, GMB24 [28]). GenBank was searched for sequences with genetic similarity to either the GMB22 or the N28353 sequences and additional subtype J *env *sequences were identified: VLGC-J1 (*env *from a virus identified in Germany), VLGC-J2 (of unknown origin) [29], the 98 BW21.17 isolate from Botswana [30] and the MBTB4, KTB147 and MBS41 isolates from DRC [31]. A phylogenetic tree was constructed as described above with these isolates, along with the six subtype J-like *env *samples from the current study (Fig. 2). All nine subtype J-like *env *sequences from the Gambia form a monophyletic cluster (with a bootstrap support of 92%) and are distinct from the DRC isolates (Fig. 2).

**Figure 3:**
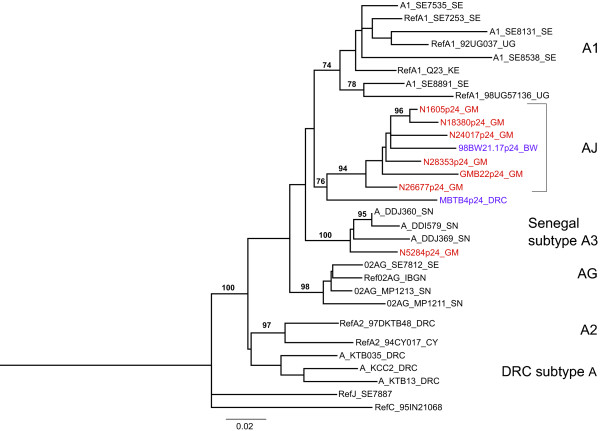
**Phylogenetic tree constructed using alignments of *gag *sequence from subtype A reference strains (denoted by prefix 'Ref'), additional subtype A1 isolates, A3 isolates from Senegal, CRF02_AG isolates and subtype A *gag *sequence from isolates with subtype J-like *env *regions**. Gambian isolates are in red, which includes an older isolate GMB22. Sequence from the non-Gambian *gag*A/envJ recombinants 98BW21.17 and MTBT4 are highlighted in purple. The cluster formed by *gag *A sequence from isolates with J-like *env *regions is boxed. One Gambian isolate (N5284_GM) falls outside this cluster. The tree was reconstructed as in Fig. 1 and bootstrap percentiles above 70% from 1000 replications (using the neighbour-joining method) are shown. The trees are rooted by outgroups formed by subtype J and C reference isolates from the Los Alamos HIV Database (2005) subtype reference set (SE7887 and 95IN21068). Branch lengths represent the number of substitutions per nucleotide sites. The tree includes the DRC isolates MTBT4, KCC2, KTBT13 and KTB035 which required the sequences to be trimmed to 623 bp. A similar tree lacking these sequences but reconstructed with a 951 bp length alignment confirmed the clustering (for the remaining sequences) although with higher bootstrap support.

The Botswana isolate was reported as a novel subtype A/J recombinant [30], although it has since been reclassified by the LAHDB as an AGJ recombinant, as parts of the genome are said to be more closely related to CRF06_AJGK than to any one isolate of subtype A or J [3]. The GMB22 and GMB24 isolates are also reported as having subtype A *gag *regions, although only *gag *sequence from GMB22 is available [28]. To test the idea that a novel recombinant is circulating in the Gambia, the *gag *p24 regions from the six novel J-like *env *isolates were sequenced and all were found to be subtype A. Furthermore the *gag *regions from the Botswana isolate 98BW21.17, GMB22 and five of the new A/J isolates form a monophyletic cluster with a bootstrap support of 94% (Fig. 3). These *gag *isolates are distinct from sub-subtype A1, A2, A3 sequences, as well as those derived from CRF02_AG isolates. One new recombinant isolate (N5284_GM) *gag *region clustered with A3 [13,14] isolates reported in surrounding Senegal, which may indicate further recombination between the novel recombinant with circulating local A3 strains. One additional isolate described in the literature, MBTB4 from DRC, is reported to have a subtype A *gag *and subtype J *env *region [31]. The subtype A *gag *phylogenetic tree was re-built including this isolate, along with three further DRC subtype A sequences (KCC2, KTB13 and KTB035), which required use of a shorter fragment length as described above. The MTBT4 isolate *gag *appears to be more closely related to subtype A *gag *regions from *gag *A/*env *J-like recombinants than other subtype A sequences (with a bootstrap support of 76%), including those from DRC (Fig. 3). Of note, the *env *region from MTBT4 clusters with the two reference J *env*s SE9173 (from an individual known to be infected in DRC) and SE92809 (bootstrap support of 98), rather than the other *env *J isolates with subtype A *gag *regions (Fig. 2).

### CRF49_cpx, a novel circulating recombinant form

**Figure 4:**
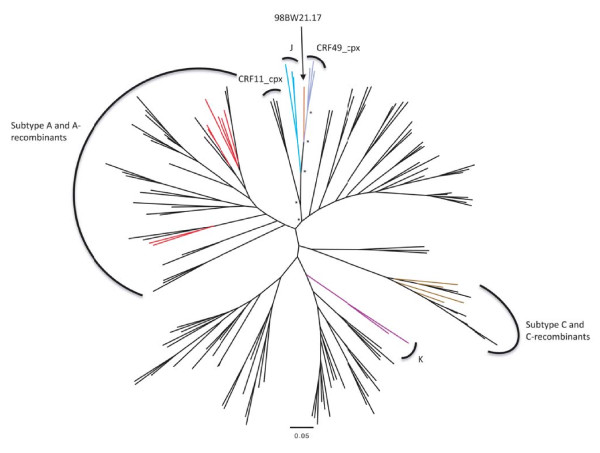
**Midpoint rooted Bayesian tree using Los Alamos 2008 subtype reference set HIV-1 full genomes, additional A3 sequences, 98BW21.17 and 3 new Gambian CRF49_cpx isolates.** Pure subtype sequences represented in the new Gambian complex recombinant are shown in color (A (red), J (turquoise), C (brown), K (purple)). Relevant nodes to the new complex recombinant, with a posterior probability of 1, are marked with *.

**Figure 5:**
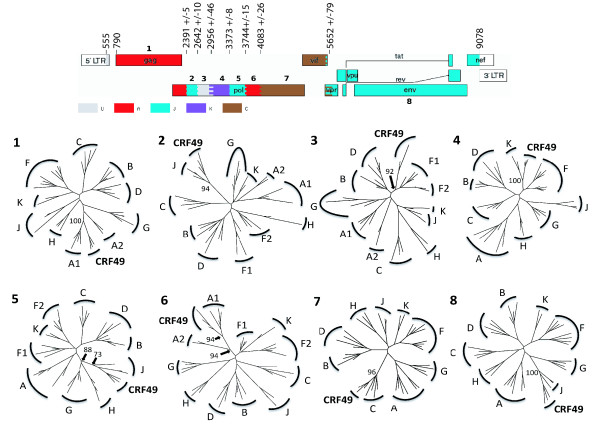
**Recombinant map of CRF49_cpx and phylogenetic trees constructed from each non-recombinant fragment**. Recombinant map of CRF49_cpx (top) drawn using the Recombinant HIV-1 Drawing Tool at LAHDB and depicting HXB2 numbering at breakpoints estimated via bootscan analysis in Simplot [22]. Below are maximum likelihood trees constructed with each non-recombinant fragment (1 to 8) showing the relationship to pure subtypes. Only bootstrap values (from 1000 replications using the neighbour-joining method) at relevant nodes to the CRF49_cpx isolates and closest pure subtype are shown for ease of presentation.

Near full-genome sequences from three of the *gag *A/*env *J-like isolates (N18380_GM, N28353_GM and N26677_GM) were generated and a phylogenetic tree constructed as described above (Fig. 4), which provided confirmation that these viruses represent a novel CRF, now named CRF49_cpx in the LAHDB. The three isolates clearly form a new cluster, separate from any currently known pure subtypes or recombinants (with a posterior probability of 1) and appear to be closely related to the Botswanan isolate 98BW21.17. Analyses of subtype recombination (as described above) revealed a complex, but consistent pattern across the three isolates (see Figs. 5, S1 and S2). In addition to the largely subtype A *gag *region and J-like *env*, a significant subtype C fragment is present in a portion of *pol*, extending through *vif *to *vpr *(which is absent in 98BW21.17), where a breakpoint with the subtype J-like fragment is found. The *pol *gene is mosaic and contains regions with similarity to subtypes A, J, K and C, as well a fragment which is not clearly defined by currently known pure subtype sequences. A phylogenetic tree constructed with this *pol *fragment (not resolved through Simplot bootscanning analysis), suggested that this region was subtype F-like (Fig. 5). Simplot and bootscan analysis [22] clearly showed a similar pattern of subtype recombination across the three isolates, although there was variation in where the exact breakpoints are (Supplementary Fig. S1 and S2), especially in the highly mosaic *pol *gene. The diversity between the three CRF49_cpx sequences may suggest that they are derived from a virus that recombined decades ago and as a great deal of evolution may have occurred since that time, many of the recombination breakpoints cannot be clearly defined. The Simplot and bootscan analysis [22] was repeated for each sequence, with inclusion of the Botswanan isolate 98BW21.17 in the reference set. This suggested that apart from the subtype C-like fragment, the CRF49_cpx sequences are more similar to 98BW21.17 than to most pure reference subtypes representing each recombinant fragment (Supplementary Fig. S3). It is possible, therefore, that CRF49_cpx originated via further recombination between a 98BW21.17-like strain and a subtype C isolate.

A careful examination of patient records was performed to determine social factors that might be associated with the CRF49_cpx viruses. There was no evidence that any of these subjects were related and there was no exclusive association with an ethnic group in this set of subjects (two Mandinka, two Manjago, one Jola and one Serahuli - see Table 1). None of these subjects were reported commercial sex workers (CSWs), one reported a blood transfusion and there were no reports of travel to the DRC or Botswana for any of the patients.

### HIV-1 subtype distribution relative to Senegal

The most recent survey from Senegal show a high prevalence of subtype C (40%), followed by CRF 02_AG (24.3%), then subtype B (18.6%) in a Senegalese cohort of men who have sex with men [32]. This distribution was different from female sex workers (FSWs) and from the general population where CRF02_AG was reported to predominate [33]. In the Senegalese FSW cohort, despite large sample numbers (328), only 2 subtype J isolates (in *env*) were reported. Because a small (385 bp) C2-V3 *env *fragment was used for subtyping [32,33], there is a concern that this might have missed detecting subtypes Js. However when the Gambian 38 samples plus the Los Alamos reference set are trimmed to the 385 bp C2-V3 region used in the Senegalese study, the six new Gambian subtype J-like *env *sequences still cluster with the reference J sequences with high bootstrap values (results not shown). If J subtypes or CRF49_cpx isolates were present in the Senegalese cohort, they would have been detected by the 385 bp C2-V3 analysis, therefore the high frequency of CRF49_cpx isolates observed in the Gambia may not extend to neighboring Senegal.

**Figure 6:**
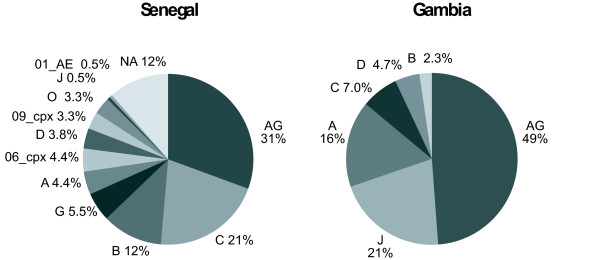
**HIV-1 subtype distribution in Senegal compared to Gambia**. The left chart shows the distribution in the 183 LAHDB sequences from Senegal >800 bp. The right chart shows the distribution in all LAHDB subtyped HIV-1 sequences from Gambia >800 bp (4 entries) plus the 38 sequences from the current work (42 entries total). Note that the CRF49_cpx viruses identified in this study were included in the J category. The frequency of selected subtypes with 95% confidence intervals (calculated using the modified Wald method) were Gambia J 21% (11.2-35.4%), Gambia CRF 02_AG 49% (34.6-63.3%); Senegal J 0.6% (< 0.01 to 3.3%), Senegal CRF 02_AG 31% (24-38%).

The geographical and subtype information in the LAHDB are gathered from investigator-supplied information. Different levels of rigor can be used to define HIV-1 subtype (e.g. the REGA HIV subtyping algorithm [34] requires a minimum of 800 bp of sequence whereas many of the LAHDB subtype designations are provided for sequences of less than 300 bp). Furthermore, of subtype designations, there can be multiple listings for the same patient and this may result in over-reporting of some subtypes. For example, for CRF02_AG, when the 840 Senegalese entries in LAHDB with reported subtype are screened for entries 800 bp or larger and the known multiple patient entries are removed, a set of 183 sequence entries remain. These 183 sequences were analysed phylogenetically, using maximum likelihood methods as described above, to generate a more stringent subtype distribution (Fig. 6, left pie). Similar criteria were applied to the 38 novel Gambian sequences from this study plus the four Gambian LAHDB entries >800 bp (Fig. 6, right pie). In this analysis, there are large differences in the frequency of the HIV-1 subtypes between the two countries (Fig 6). This could be due to cultural differences, or to differences in the age and extent of the epidemic in each country. In addition, the Senegalese data are dominated by sequences derived from specific cohorts (MSM, CSW) while the Gambia data (mostly derived from the current study) come from random selection of patients attending a GUM clinic; such differences in the patient composition could results in the large differences in the subtype distribution.

## Conclusions

Information on the diversity of HIV-1 in the Gambia is currently lacking and the current study has attempted to address this gap by generating full-length HIV-1 *env *sequences from 38 local HIV-1 isolates. Documentation of the ongoing HIV-1 epidemic and sequence data from West Africa is important for several reasons. In a region where HIV-1 diversity is higher than in many other parts of sub-Saharan Africa, such information is required to maintain accurate viral diagnostics and sensitive viral load assays. HIV-1 subtypes may differ biologically in areas such as viral fitness [35,36] and co-receptor usage (e.g. likelihood of switch from R5 to X4 usage) [37,38]. These may in turn translate into higher risk of disease progression in certain subtypes and recombinant viruses could also have certain advantages over their parent strains. Studies in East Africa, using both prevalent and incident infections, have shown a higher risk of progression to AIDS and AIDS-related death in subtype D (and inter-subtype recombinant) -infected individuals when compared to subtype A-infected patients [39,40]; even following adjustment for baseline viral load [41]. A Senegalese study supports the notion that non-A subtype infections progress faster than subtype A infections [42], although outcomes in CRF02_AG infected individuals appear to be no worse compared to non-AG infections [43]; despite the rise of this circulating recombinant form (CRF) in West Africa and *in vitro *data suggesting enhanced viral fitness [35]. With the increasing availability of anti-retroviral therapy (ART) in West Africa, it is also important to consider potential differences between HIV-1 subtypes in drug resistance pathways and the ease with which resistance appears due to naturally occurring polymorphisms (e.g. the development of K65R in subtype C infections) [44,45]. Such findings would clearly have implications for local ART regimes and choice of 2^nd ^line drugs. Finally, local sequence data are important in the design of potential immunogens for future prophylactic and therapeutic HIV-1 vaccines, although the greater diversity in West Africa makes this daunting task even more challenging in this subregion. Mosaic vaccine strategies [4,5] may overcome this barrier and documentation of new CRFs and accurate representation of global sequence diversity is essential for these strategies.

While the majority prevalence of subtype A and CRF02_AG in the new set of HIV-1 isolates is consistent with data from other West African countries, the identification of 6 isolates of a novel recombinant, CRF49_cpx, in the 38 isolates was surprising and unique to the Gambia. These six infected individuals were epidemiologically unlinked and *env *sequence from these viruses cluster with three previously described Gambian subtype J-like *env *sequences. Thus, all nine isolates are likely to represent the novel HIV-1 CRF49_cpx. Full genome sequence from the Botswanan isolate (98BW21.17) [30] is closely linked to the Gambian isolates in phylogenetic analyses (more so than to any other virus currently in the LAHDB). Due to the limited number of patients examined, it is difficult to predict the importance of CRF49_cpx in the Gambian HIV-1 epidemic. Although some criteria were imposed in sample selection, within both patient groups (CD4 >/= 28% at first presentation and recently diagnosed and commenced antiretroviral therapy) selection was randomized. There is good reason to believe therefore, that CRF49_cpx may represent a reasonable proportion of the HIV-1 infections in the Gambia. Further studies are important to clarify its prevalence (including changes over time), the contribution to new infections in recent years and the disease potential relative to other local subtypes.

## Competing interests

The authors declare that they have no competing interests.

## Authors' contributions

TdS participated in all parts of the study, co-supervised the work, was responsible for *env *and *gag *sequencing, participated in the sequence and phylogenetic analysis and participated in writing the manuscript, RT was responsible for amplifying and sequencing most of the *envs *and helped with the initial analysis of the sequences, SH provided detailed phylogenetic support and assisted in writing the manuscript, RTr assisted in generating near full-genome sequence from the three described CRF49_cpx isolates, CvT provided database analysis, organized the patient data and participated in writing the manuscript, CO participated in designing the sequencing strategy and methods and assisted in the initial analysis of the sequences, BF helped interpret the recombination and phylogenetic analyses of CRF49_cpx and assisted in writing the manuscript, HW participated in the design of the study, provided virological support and participated in writing the manuscript, SR-J participated in the design of the study, provided virological support and participated in writing the manuscript, AJ provided virological support and participated in writing the manuscript, MC participated in all parts of the study, co-supervised the work, assisted in the sequence analysis and phylogenetic analysis and participated in writing the manuscript.

## Supplementary Material

Additional file 1 **Figure S1 - Bootscan analyses of CRF49_cpx isolates N18380_GM (a), N26677_GM (b) and N28353_GM (c) performed with Simplot **[22]**and including HIV-1 subtypes A through K**. Alignment was gap stripped. Sliding window was set to 400 bp with increments set to 50 bp. Bootscanning was performed by neighbour-joining tree construction model, using the Kimura (two-parameter) distance model and 100 bootstrap replicates for each sliding window. Transition/traversion ratio was set to 2.0. Click here for file

Additional file 2 **Figure S2 - Simplot analyses of CRF49_cpx isolates N18380_GM (a), N26677_GM (b) and N28353_GM (c) **[22]** and including HIV-1 subtypes A through K**. Alignment was gap stripped. Sliding window was set to 400 bp with increments set to 50 bp. Bootscanning was performed by neighbour-joining tree construction model, using the Kimura (two-parameter) distance model and 100 bootstrap replicates for each sliding window. Transition/traversion ratio was set to 2.0. Click here for file

Additional file 3 **Figure S3 - Simplot analyses of CRF49_cpx isolates N18380_GM (a), N26677_GM (b) and N28353_GM (c) **[22]**and including HIV-1 subtypes A through K and Botswana isolate 98BW21.17**. Alignment was gap stripped. Sliding window was set to 400 bp with increments set to 50 bp. Bootscanning was performed by neighbour-joining tree construction model, using the Kimura (two-parameter) distance model and 100 bootstrap replicates for each sliding window. Transition/traversion ratio was set to 2.0. Click here for file
